# Burden of kidney failure from atheroembolic disease and association with survival in people receiving dialysis in Australia and New Zealand: a multi-centre registry study

**DOI:** 10.1186/s12882-021-02604-7

**Published:** 2021-12-02

**Authors:** Tahira Scott, Isabelle Ethier, Carmel Hawley, Elaine M. Pascoe, Andrea K. Viecelli, Arnold Ng, Yeoungjee Cho, David W. Johnson

**Affiliations:** 1 Department of Nephrology, Level 2, ARTS Building, Princess Alexandra Hospital, 199 Ipswich Road, Woolloongabba, Brisbane, QLD 4102 Australia; 2 School of Medicine, University of Queensland, Brisbane, Australia; 3 Division of Nephrology, Centre Hospitalier de l’Université de Montréal, Montréal, Canada; 4 Australia and New Zealand Dialysis and Transplant (ANZDATA) Registry, Adelaide, Australia; 5 Australasian Kidney Trials Network, University of Queensland, Brisbane, Australia; 6 Translational Research Institute, Brisbane, Australia; 7 Department of Cardiology, Princess Alexandra Hospital, Brisbane, Australia

**Keywords:** Atheroembolic disease, Dialysis, Kidney failure, Outcome, Registry, Survival

## Abstract

**Background:**

Cardiovascular disease is a leading cause of mortality in kidney failure (KF). Patients with KF from atheroembolic disease are at higher risk of cardiovascular disease than other causes of KF. This study aimed to determine survival on dialysis for patients with KF from atheroembolic disease compared with other causes of KF.

**Methods:**

All adults (≥ 18 years) with KF initiating dialysis as the first kidney replacement therapy between 1 January 1990 and 31 December 2017 according to the Australia and New Zealand Dialysis and Transplant registry were included. Patients were grouped into either: KF from atheroembolic disease and all other causes of KF. Survival outcomes were assessed by the Kaplan-Meier method and Cox regression analysis adjusted for patient-related characteristics.

**Results:**

Among 65,266 people on dialysis during the study period, 334 (0.5%) patients had KF from atheroembolic disease. A decreasing annual incidence of KF from atheroembolic disease was observed from 2008 onwards. Individuals with KF from atheroembolic disease demonstrated worse survival on dialysis compared to those with other causes of KF (HR 1.80, 95% confidence interval [CI] 1.61–2.03). The respective one- and five-year survival rates were 77 and 23% for KF from atheroembolic disease and 88 and 47% for other causes of KF. After adjustment for patient characteristics, KF from atheroembolic disease was not associated with increased patient mortality (adjusted HR 0.93 95% CI 0.82–1.05).

**Conclusions:**

Survival outcomes on dialysis are worse for individuals with KF from atheroembolic disease compared to those with other causes of KF, probably due to patient demographics and higher comorbidity.

## Introduction

Patients with kidney failure (KF) experience a higher mortality rate compared to the general population across all age groups. A leading cause of morbidity and mortality while on dialysis is atherosclerotic cardiovascular disease, contributing up to 50% of all-cause mortality [1]. Atherosclerotic cardiovascular disease also predisposes to an increased incidence of KF from atheroembolic disease of the kidney. Atheroembolic disease is a multisystem entity, whose aetiology is often iatrogenic after cardiac surgery, angiography, anticoagulation and thrombolysis but can occur spontaneously [2]. These precipitating events lead to the dislodgment of cholesterol emboli from the aorta to the kidney vasculature, leading to ischemia and KF [3].

The incidence of KF from atheroembolic disease is unknown but the prevalence is thought to be 1% [4, 5]. There is limited data published on the morbidity and mortality of patients with KF from atheroembolic disease on dialysis. Several cohort studies suggest that clinical outcomes are worse for atheroembolic kidney disease than for other forms of kidney disease, including high rates of acute dialysis (28–61%) [6–8], maintenance dialysis (25–35%) [6, 7] and one-year mortality (64–87%) [3]. There are also no previous studies to our knowledge, that outlines the rate of transplantation in those with KF from atheroembolic disease.

This study aimed to evaluate patient survival on dialysis, kidney function recovery and rate of kidney transplantation in those with KF from atheroembolic disease compared with other forms of KF in the Australian and New Zealand populations, using data from the Australia and New Zealand Dialysis and Transplant (ANZDATA) registry.

## Material and methods

### Patient population

All incident adults (≥18 years) with KF enrolled in the ANZDATA registry who commenced kidney replacement therapy (KRT) between 15 May 1963 and 31 December 2017 were considered for inclusion. A contemporary cohort of patients commencing KRT between 1 January 1990 and 31 December 2017, consisting of only patients who started KRT with dialysis was included for the final analysis, as there were only four cases of KF from atheroembolic disease before 1990.

Patients were categorised into two groups according to the cause of their primary kidney disease (atheroembolic disease and all other causes of KF), as recorded in the ANZDATA registry. The cause of KF was determined by each patient’s primary nephrologist based on clinical picture, laboratory investigations and kidney biopsy, if available. This study used de-identified data from the ANZDATA registry with permission granted by the ANZDATA Executive. Ethical approval for the use of registry data was obtained from the Princess Alexandra Hospital Human Research Ethics Committee (LNR/2019/QMS/50454). The study was conducted in accordance with STROBE guidelines [9].

### Study outcomes

The primary outcome of this study was patient survival on dialysis, defined as death on dialysis (censored for kidney function recovery, loss to follow-up, kidney transplantation and end of study). Secondary outcomes examined were the annual proportion of KF attributed to atheroembolic disease, patient characteristics associated with KF from atheroembolic disease and recovery of kidney function defined as completion of dialysis therapy documented by the treating nephrologist. The onset of recovery was defined as the date of the last dialysis treatment.

### Statistical analysis

Patient characteristics were expressed as frequency and percentages for categorical variables and median (interquartile range) for non-normally distributed continuous variables. Chi-squared (for categorical variables) and Wilcoxon sign rank test (for non-normally distributed continuous variables) tests were used to assess differences between cohorts.

Time-to-event analyses of patients with KF from atheroembolic disease versus all other KF causes were evaluated by Kaplan-Meier survival analysis using the log-rank test. Univariable and multivariable Cox proportional hazard survival analyses were completed with the covariates of disease group (atheroembolic vs all other KF), age, sex, ethnicity (Caucasian vs other), sub-era (1990–1998, 1999–2007 and 2008–2017), kidney replacement treatment (KRT) modality, late referral (defined as commencement of dialysis within 3 months of referral to a nephrologist), smoking status (current vs never/former), body mass index, comorbidities (diabetes mellitus, coronary artery disease, peripheral vascular disease, chronic lung disease and cerebrovascular disease) and country (Australia vs New Zealand). The proportionality assumption was examined visually with log-log survival plots. There were no significant first-order interactions identified. As a sensitivity analysis, a propensity score was generated using multivariable logistic regression including covariates in the multivariable Cox model to conduct 1:3 matched analysis (matched using nearest neighbor, no replacement approach). Statistical analysis was performed by Stata/IC version 16.1 (StataCorp, College Station, TX) software packages. *P* values < 0.05 were considered statistically significant.

## Results

### Patient characteristics

**Fig. 1:**
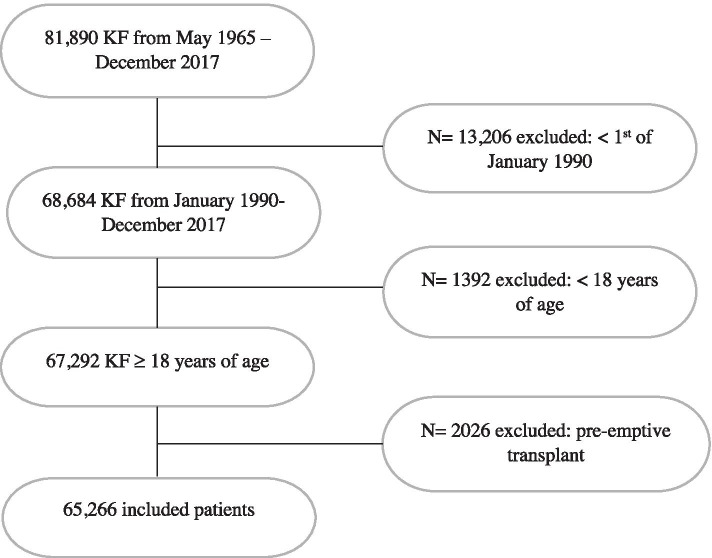
Study flow diagram. KF = kidney failure

Between January 1990 and December 2017**,** 65,266 patients started dialysis, including 334 (0.5%) patients with atheroembolic disease as the cause of KF (Fig. 1). The baseline characteristics are displayed in Table 1. Patients with KF from atheroembolic disease were older at the commencement of KRT (72 vs 61 years), predominantly male (81% vs 60%), Caucasian (96% vs 70%), referred late (51% vs 21%) and more likely to have coronary artery disease (80% vs 31%), peripheral vascular disease (59% vs 18%), chronic lung disease (23% vs 12%) and cerebrovascular disease (32% vs 11%). Missing data were less than 5% for all variables except for late referral (6%) and kidney biopsy status (8.6%; Table 1).

**Table 1:** Baseline patient characteristics of the complete cohorts

Characteristics	All KF	KF from atheroembolic disease	KF all other causes	***P***-value
***N*=**	**65,266**	**334**	**64,932**	
**Age at cohort entry (years)**	59 (49–71)	72 (68–76)	61 (49–71)	**< 0.001**
**Male sex**	39,235 (60%)	270 (81%)	38,965 (60%)	**< 0.001**
**Primary kidney disease**				**< 0.001**
Atheroembolic		334 (100%)		
Diabetic nephropathy			21,811 (33%)	
Glomerulonephritis			15,879 (24%)	
Reflux nephropathy			1890 (3%)	
Polycystic disease			3944 (6%)	
Hypertension			8339 (13%)	
Other			8866 (14%)	
Uncertain			3716 (6%)	
Not reported			487 (1%)	
**Ethnicity**				**< 0.001**
Caucasian	45,720 (70%)	321 (96%)	45,399 (70%)	
All Other	19,544 (30%)	13 (4%)	19,531 (30%)	
**BMI (kg/m**^**2**^**)**^**a**^				**< 0.001**
< 18.5	2165 (3%)	16 (5%)	2149 (3%)	
18.5–24.9	22,195 (35%)	174 (54%)	22,021 (35%)	
25–29.9	19,875 (32%)	102 (31%)	19,773 (32%)	
≥ 30	18,702 (30%)	33 (10%)	18,669 (30%)	
**Comorbidities**^**b**^				
Diabetes mellitus	28,147 (44%)	76 (23%)	36,141 (56%)	**< 0.001**
CAD	19,920 (31%)	265 (80%)	19,920 (31%)	**< 0.001**
PVD	11,653 (18%)	195 (59%)	11,458 (18%)	**< 0.001**
Chronic lung disease	7986 (12%)	77 (23%)	7909 (12%)	**< 0.001**
Cerebrovascular disease	7030 (11%)	104 (32%)	6926 (11%)	**< 0.001**
**Current smoking**^**c**^	8532 (13%)	43 (13%)	8489 (13%)	**0.85**
**Country at cohort entry**				**< 0.001**
Australia	53,697 (83%)	318 (95%)	53,649 (83%)	
New Zealand	11,299 (17%)	16 (5%)	11,283 (17%)	
**Late referral**^**d**^	13,127 (21%)	154 (51%)	12,973 (21%)	**< 0.001**
**Kidney biopsy**^**e**^	18,903 (29%)	92 (28%)	18,811 (29%)	**0.92**
**Dialysis Era**				
1990–1998	13,282 (20%)	96 (29%)	13,186 (20%)	**< 0.001**
1999–2007	21,521 (33%)	174 (52%)	21,347 (32%)	
2008–2017	30,463 (47%)	64 (19%)	30,399 (46%)	
**First dialysis modality**				**< 0.001**
Facility HD	46,540 (71%)	275 (82%)	46,265 (71%)	
PD	18,441 (28%)	59 (18%)	18,382 (28%)	
HHD	285 (0.5%)	0	285 (0.44%)	

Values are expressed as frequency (percentage) for categorical variables, mean ± standard deviation for normally distributed continuous variables, and median (interquartile range) for non-normally distributed continuous variables

*BMI* Body Mass Index, *CAD* Coronary Artery Disease, *CVD* Composite Cardiovascular Disease; *HD* Hemodialysis, *HHD* Home Hemodialysis, *KF* Kidney Failure, *N* Number, *PD* Peritoneal Dialysis, *PVD* Peripheral Vascular Disease

^a^Data on BMI were missing for 4% of patients

^b^Data on comorbidities were missing for less than 2% of patients

^c^Data on smoking status were missing for 3% of patients

^d^Data on late referral were missing for 6% of patients

^e^Data on Kidney Biopsy were missing for 8.6% of patients

### The burden of Atheroembolic disease

**Fig. 2:**
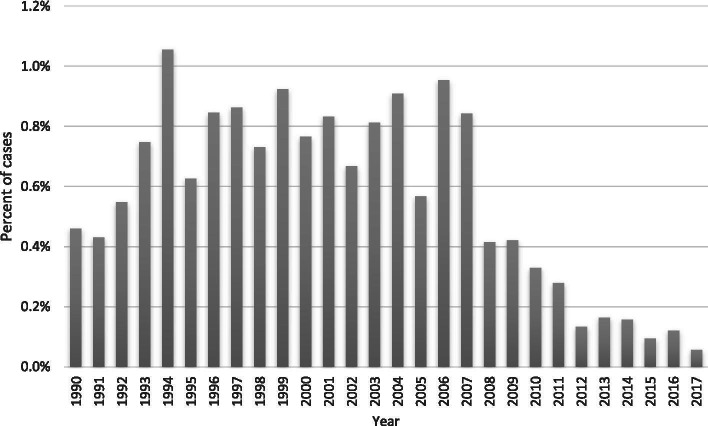
Annual incidence of kidney failure from atheroembolic disease expressed as a percentage per year of total cases of all kidney failure

The incidence of KF from atheroembolic disease was low compared to other causes of KF including those attributable to renovascular disease (*n* = 472, 0.7%) and hypertension (*n* = 8673, 13%). The burden of KF from atheroembolic disease was highest from 1999 to 2007, representing 52% of the cases, and decreased substantially from 2008 to 2017 (Table 1 and Fig. 2).

### Patient survival on Dialysis

During the study period, there were 34,571 deaths, with 283 (0.8%) among those with KF from atheroembolic disease (1037 years at risk) and 34,288 (99.2%) from KF from other causes (mortality incidence rate of 272.7 per 1000 person-years for KF from atheroembolic disease vs 152 per 1000 person-years for KF from other causes).

The median survival of those with KF from atheroembolic disease (2.87 years, 95% confidence interval [CI] 2.53–3.36) was shorter than for KF from all other causes (4.67 years, 95% CI 4.61–4.72). Respective survival rates after starting KRT were 77% versus 88% at 1 year and 23% versus 47% at 5 years. The causes of death on dialysis were comparable between the two groups. Cardiac death was the most common (35% vs. 37%), followed by dialysis withdrawal (32% vs 29%), infection (11.5% vs 11.5%), vascular (10% vs 8.6%) and other (12% vs 13%).

**Fig. 3:**
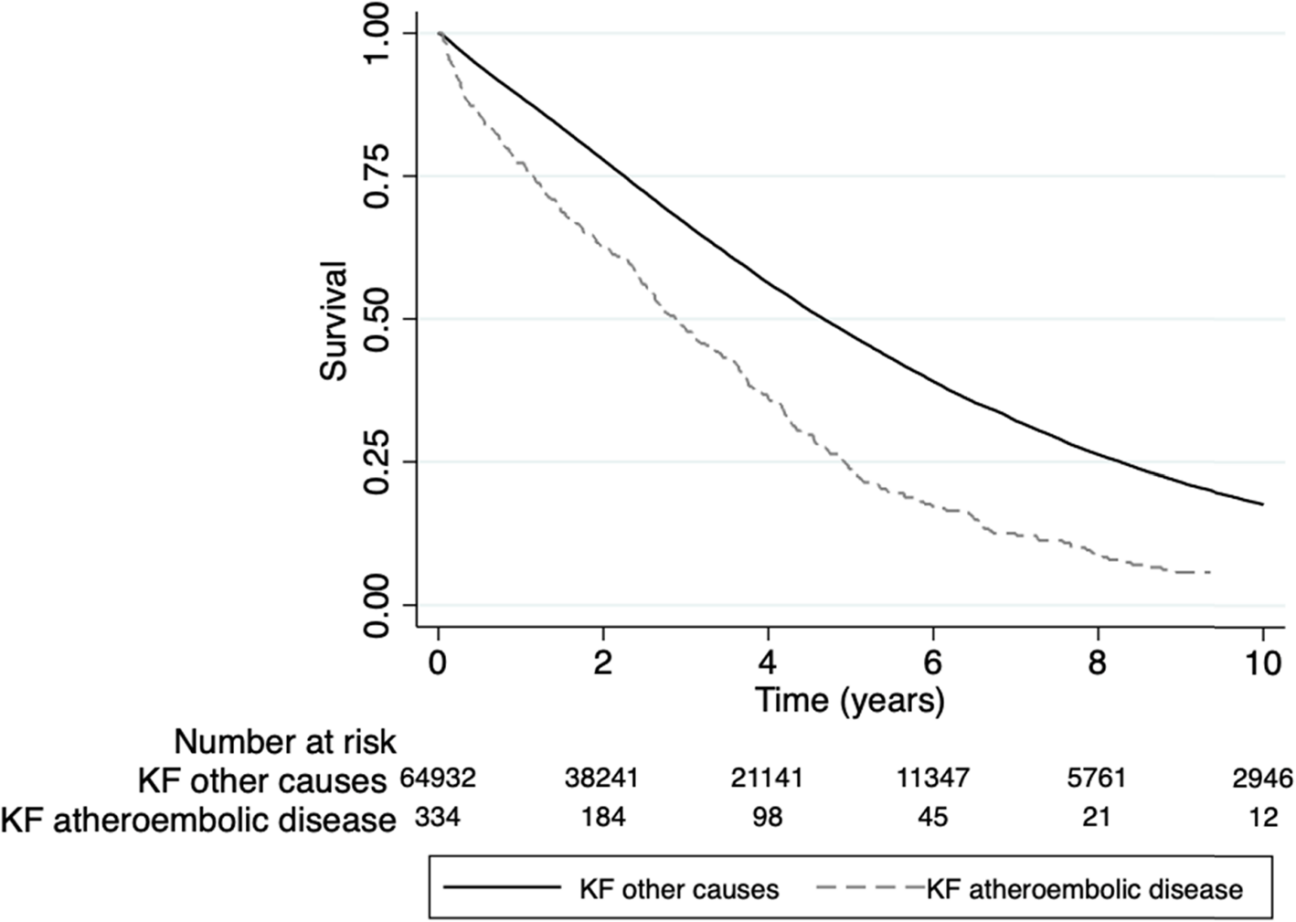
Survival on dialysis comparing KF from other causes to KF from atheroembolic disease, unadjusted. Dialysis Survival overall log-rank of atheroembolic disease KF vs other KF, *p*-values < 0.001. KF = Kidney Failure

In univariable Cox proportional hazard model analysis, KF from atheroembolic disease was associated with an increased risk of death (Fig. 3) (hazard ratio [HR] 1.80, 95% CI 1.61–2.03). However, after adjustment for patient characteristics, this association was no longer observed (adjusted HR [aHR] 0.93, 95% CI 0.82–1.05). In the multivariable model, the risk of mortality remained significant for age per decade (aHR 1.43, 95% CI 1.42–1.45), BMI < 18.5 kg/m^2^ (aHR 1.43, 95% CI 1.35–1.52), late referral (aHR 1.23, 95% CI 1.20–1.26), diabetes mellitus (aHR 1.46, 95% CI 1.43–1.50), coronary artery disease (aHR 1.30, 95% CI 1.28–1.34), peripheral vascular disease (aHR 1.32, 95% CI 1.28–1.35), chronic lung disease (aHR 1.30, 95% CI 1.26–1.34), cerebrovascular disease (aHR 1.20, 95% CI 1.17–1.24), current smoking (aHR 1.26, 95% CI 1.22–1.30), peritoneal dialysis (aHR 1.03, 95% CI 1.01–1.06), dialysis era 1990–1998 (aHR 1.30, 95% CI 1.26–1.35) and New Zealand residence (adjusted HR 1.44, 95% CI 1.39–1.48) (Table 2).

**Table 2:** Univariable and multivariable Cox proportional hazard model analyses for KF from all other and Atheroembolic KF

Characteristics	UNIVARIATE			MULTIVARIATE		
	HR	95% CI	***p***-value	HR	95% CI	***p***-value
All other KF	Ref			Ref		
KF from Atheroembolic Disease	1.80	1.61–2.03	< 0.001	0.93	0.82–1.05	0.24
Age per decade at cohort entry	1.41	1.40–1.43	< 0.001	1.43	1.42–1.45	< 0.001
Male sex	1.04	1.01–1.06	< 0.001	0.95	093–0.97	< 0.001
Primary kidney disease			< 0.001			
Diabetic nephropathy	Ref					
Glomerulonephritis	0.56	0.55–0.58	< 0.001			
Reflux nephropathy	0.39	0.35–0.42	< 0.001			
Polycystic disease	0.42	0.40–0.45	< 0.001			
Hypertension	1.13	1.09–1.16	< 0.001			
Other	1.04	1.01–1.07	< 0.004			
Racial origin			< 0.001			< 0.001
Caucasian	Ref			Ref		
Other	0.75	0.73–0.76	< 0.001	0.83	0.81–0.85	< 0.001
BMI (kg/m^2^)			< 0.001			< 0.001
< 18.5	1.25	1.18–1.32	< 0.001	1.43	1.35–1.52	< 0.001
18.5–24.9	Ref					< 0.001
25–29.9	0.94	0.92–0.97	< 0.001	0.88	0.86–0.90	< 0.001
≥ 30	0.84	0.82–0.86	< 0.001	0.85	0.82–0.87	< 0.001
Late referral	1.17	1.14–1.20	< 0.001	1.23	1.20–1.26	< 0.001
Comorbidities						
Diabetes	1.38	1.35–1.41	< 0.001	1.46	1.43–1.50	< 0.001
CAD	1.86	1.82–1.90	< 0.001	1.30	1.28–1.34	< 0.001
PVD	1.89	1.85–1.94	< 0.001	1.32	1.28–1.35	< 0.001
Chronic lung disease	1.64	1.60–1.69	< 0.001	1.30	1.26–1.34	< 0.001
Cerebrovascular disease	1.74	1.65–1.75	< 0.001	1.20	1.17–1.24	< 0.001
Current smoking	0.97	0.94–1.00	0.06	1.26	1.22–1.30	< 0.001
Dialysis eras (years)			< 0.001			< 0.001
1990–1998	1.22	1.19–1.26	< 0.001	1.30	1.26–1.35	< 0.001
1999–2007	Ref					
2008–2017	0.88	0.86–0.90	< 0.001	0.85	0.83–0.87	< 0.001
Modality			< 0.001			< 0.001
HD	Ref					
HHD	0.22	0.14–0.35	< 0.001	0.31	0.19–0.50	< 0.001
PD	1.04	1.02–1.07	< 0.001	1.03	1.01–1.06	0.01
Country						
Australia	Ref					
New Zealand	1.09	1.06–1.12	< 0.001	1.44	1.39–1.48	< 0.001

Multivariable analysis adjusted for: disease group (atheroembolic vs all other KF), age, gender, racial origin (Caucasian vs other), sub-era, kidney replacement modality, late referral, current smoking, body mass index, comorbidities at entry (diabetes mellitus, coronary artery disease, peripheral vascular disease, chronic lung disease and cerebrovascular disease) and country (Australia vs New Zealand)

*95% CI* 95% confidence interval, *BMI* Body Mass Index, *CAD* Coronary Artery Disease, *CVD* Composite Cardiovascular Disease, *HD* Hemodialysis, *HHD* Home Hemodialysis, *HR* Hazard Ratio, *N* Number, *PD* Peritoneal Dialysis, *PVD* Peripheral Vascular Disease, *Ref* Reference

### Propensity score matching

In the propensity score-based 1:3 matched analysis, patient survival was again comparable between patients with KF from atheroembolic disease and other causes (HR = 1.02, 95% CI 0.89–1.18, *p* = 0.68).

### Kidney recovery

There were 31 patients with KF from atheroembolic disease who had kidney function recovery and therefore were independent of dialysis (*n* = 2 [6%] < 30 days on dialysis and *n* = 29 [94%] after > 90 days on dialysis) versus 1368 patients with KF from other causes (*n* = 190 [14%] < 30 days from dialysis, *n* = 314 [23%] after 30–90 days on dialysis and *n* = 864 [63%] after > 90 days on dialysis, Table 3).

**Table 3:** End of study event outcomes

	All	KF from atheroembolic disease	KF all other causes
***n*=**	**65,266**	**334**	**64,932**
Death on dialysis	34,571	283 (84.7%)	34,288 (52.8%)
Total kidney recovery	1399	31 (9.3%)	1368 (2.1%)
*Kidney recovery < 30 days*		2 (6.5%)	190 (14%)
*Kidney recovery 30–89 days*		0	314 (23%)
*Kidney recovery > 90 days*		29 (93.5%)	864 (63.2%)
Transplant	15,049	5 (1.5%)	15,044 (23%)
Loss to follow up	124	0	124 (19%)
End of study	14,123	15 (4.5%)	14,108 (21.7%)

*KF* Kidney failure

### Transplant

Patients with KF from atheroembolic disease were significantly less likely to receive a kidney transplant (*n* = 5) throughout the study period compared to those with other causes of KF (*n* = 15,044; 1.5% vs 23%, *P* = 0.001).

## Discussion

This is the largest multicenter study to date comparing dialysis survival outcomes of KF from atheroembolic disease to all other causes of KF. It showed those with KF from atheroembolic disease were less likely to survive on dialysis, with the lead cause of death being cardiovascular disease followed by dialysis withdrawal. However, after adjustment for demographic factors and comorbidities, those with KF from atheroembolic disease no longer had lower survival on dialysis, suggesting that the accompanying risk factors for atheroembolic kidney disease predisposed to death rather than the disease itself. In this study, those with KF from atheroembolic disease were more likely to become independent from dialysis after a longer duration on KRT of more than 90 days and had a lower rate of transplantation in comparison to all other KF.

We observed a 1-year survival rate of 77% in patients with KF from atheroembolic disease, which appears to be comparable to that of previous studies of 82–87% [6, 7]. The median survival of 2.87 years was worse than the findings of a single centre observational study which reported a median survival of 4.08 years. Besides the smaller cohort size of 95 patients, the other main disparity between this cohort study and our study that may explain our inferior survival rate was a lower rate of diabetes mellitus of 15% (versus 23%) and their cohort was mostly patients acutely initiated on dialysis, of whom only 24% remained on maintenance dialysis [6]. Lastly, the above two studies only reviewed outcomes in Atheroembolic KF and did not compare to other causes of KF. Cardiovascular disease was the leading cause of death in our study, similar to other reports [3]. This is unsurprising given the atherosclerotic background of this population.

Although KF due to atheroembolic disease was associated with higher mortality in univariable Cox regression compared to other causes of KF, this association was no longer significant after adjustment for other patient characteristics. In multivariable Cox regression models, older age, late referral to nephrologist care, diabetes mellitus, coronary artery disease, peripheral vascular disease, chronic lung disease, cerebrovascular disease, current smoking, peritoneal dialysis, the dialysis era 1990–1998 and being from New Zealand were associated with significantly higher mortality. Aggressive management of predisposing comorbidities and earlier referral to nephrology care could represent potential target areas for management in this population. However, it could be postulated that the higher mortality associated with late referral might be related to the indication for angiography leading to atheroembolic disease, independent of kidney function. Furthermore, a late referral is inevitable in many cases of KF due to atheroembolic disease, given that it is iatrogenic in greater than 75% of cases in the setting of unavoidable interventions, frequently in those with pre-existing CKD [10].

In this study, atheroembolic disease represented 0.5% of all causes of KF in patients initiating dialysis in Australia and New Zealand between 1990 and 2017. This is consistent with previous studies reporting a prevalence of atheroembolic disease on kidney biopsies around 1% [7, 8]. An interesting trend identified in the present study was that the annual incidence of cases of KF from atheroembolic disease has fallen since 2008, with an overall lower incidence of cases in the dialysis era of 2008–2017 (19%) versus other eras (1990–1998, 29% & 1999–2007, 52%). We hypothesize that this observation was related to a temporal change in Australian cardiology practice with higher uptake of the radial approach versus femoral approach for coronary angiography across this period [11]. This practice change is supported by the National Heart Foundation guidelines recommending the radial approach as the preferred access for percutaneous intervention [12]. Femoral intra-arterial access is associated with higher rates of acute kidney injury compared with the radial approach [2] and presumably therefore also with more atheroembolic disease, documented to cause up to 78% of atheroembolic kidney disease [9], due to the catheter traversing the abdominal aorta dislodging atherosclerotic plaque into the renal arteries [10]. Another potential explanation for the declining incidence seen in our study is a growing tendency by nephrologists towards offering conservative care rather than dialysis to patients who are older and more co-morbid, with ANZDATA only capturing patients who start KRT. There has been a steady growth of dedicated kidney supportive care services in nephrology units across Australia and New Zealand [13], although the referral patterns and clinical uptake of these services have not been directly studied. Lastly, KF from atheroembolic disease may also be relatively under-diagnosed and under-reported in patients with other CKD risk factors, such as diabetes and hypertension, who are less likely to undergo kidney biopsy, as exemplified by a low overall biopsy rate of 29% within our study.

There was minimal kidney function recovery for patients with KF attributable to atheroembolic disease in our study, in contrast to other studies reporting rates of recovery and freedom off dialysis ranging from 21 to 39% [3]. This result may be explained by the structure of ANZDATA registry which only enrolls patients anticipating chronic dialysis and does not include patients with acute but temporary kidney failure. Despite the low recovery rate, those with KF from atheroembolic disease were more likely than patients with other aetiologies to have kidney function recovery after a dialysis duration of 90 days or more. A proposed mechanism could be stabilization of aortic cholesterol plaques and continued regression of embolized crystals after the precipitating event and ostensible commencement of statin therapy. Statin therapy is known to stabilise and regress cholesterol plaques and has been documented to improve kidney outcomes in atheroembolic disease [6, 14].

Overall, there were low rates of transplantation, with no pre-emptive kidney transplants for those with KF from atheroembolic disease. The small number of kidney transplantations prevented meaningful analysis of outcomes in comparison to other causes of KF undergoing transplantation. The low transplantation rate is plausibly related to the patient’s profiles observed in our study, with those with KF from atheroembolic disease being older Caucasian men with comorbidities such as coronary artery disease, peripheral vascular disease, chronic lung disease and cerebrovascular disease. This older population with a risk profile would typically be considered ineligible or unfavourable for transplantation. This is further exemplified by the observed low uptake of home therapies within the cohort. To our knowledge, this is the first study within the literature that has looked at transplantation and home therapies uptake in KF from atheroembolic disease.

The strengths of our study were the large sample size (*n* = 65,266)**,** long follow-up duration (total 225,432 patient-years), sufficient event numbers to enable assessment of mortality and comprehensive capture of data on all patients starting KRT in Australia and New Zealand. One of the limitations of the study was that ANZDATA registry does not collect information on those with stage 5 CKD who elected to receive conservative care rather than KRT, such that the absence of this information would reduce the overall number of analysed cases and may have led to ascertainment bias if the uptake of KRT differed between atheroembolic kidney disease and other forms of KF. Furthermore, there was a limited depth of data collection, such that the possibility of residual confounding could not be excluded. There was also the potential for coding bias with respect to nephrologists and their diagnoses of KF from atheroembolic disease.

In conclusion, this study demonstrates that patients with KF from atheroembolic disease have a higher mortality rate on dialysis in comparison to those with KF from other aetiologies, which was no longer observed after adjustments for patient demographic characteristics and comorbidities. It also demonstrates a temporal trend in the reduction of the annual incidence of KF from atheroembolic disease. Further research is warranted to determine whether this temporal trend reflects changes in angiographic practices, changes in vascular disease prevalence or other factors.

## Data Availability

The dataset analysed during the study is available through the ANZDATA registry and can be requested by completing a data request and emailing requests@anzdata.org.au.
